# Supplementary material for: Tibetan Plateau grasslands might increase sequestration of microbial necromass carbon under future warming
Source: Commun Biol. 2024 Jun 4;7:686. doi: 10.1038/s42003-024-06396-y (PMC11150409; doi:10.1038/s42003-024-06396-y)
Supplement: Supplementary file 2 — Supplementary Information [file 42003_2024_6396_MOESM2_ESM.pdf]

Tibetan Plateau grasslands might increase sequestration of microbial necromass carbon under future warming

Qinwei Zhang[1,2†], Xianke Chen[1,2†], Xiaorong, Zhou[1,2], Xin Nie[1,2], Guohua Liu[1,2], Guoqiang Zhuang[1,2,3], Guodong Zheng[4], Danielle Fortin[5], Anzhou Ma[1,2*]

[1]Research Centre for Eco-Environmental Sciences, Chinese Academy of Sciences, Beijing, 100085, China.

[2]University of Chinese Academy of Sciences, Beijing, 100049, China.

[3]Binzhou Institute of Technology, Binzhou, 256600, China.

[4]School of Environmental Studies, China University of Geosciences, Wuhan, 430078, China.

[5]Department of Geology, University of Ottawa, Ottawa, K1N6N5, Canada.

[*]Corresponding author:

Anzhou Ma

Email: azma@rcees.ac.cn

Phone: 86-010-6284-9156

[†]These authors contributed equally: Qinwei Zhang, Xianke Chen.

This PDF file includes:

Supplementary Figures 1-7

Supplementary Tables 1-3

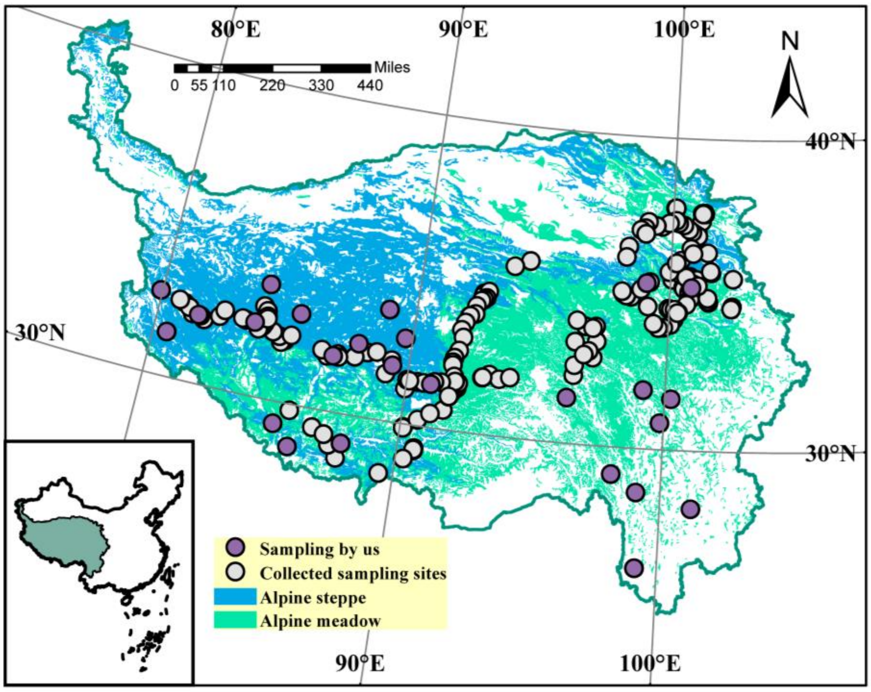

Fig. S1 Distribution of sampling sites across the Tibetan Plateau.

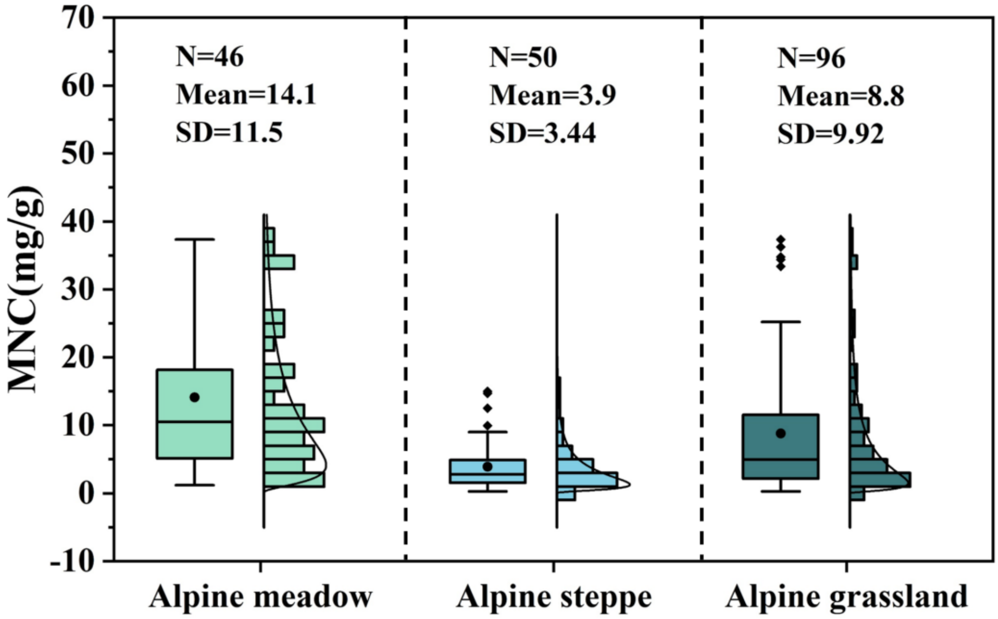

Fig. S2 Comparison of MNC between alpine meadow, alpine steppe and the whole alpine grassland. The horizontal line and square black dots in each box represent the median and mean, respectively. Alpine grassland consists of alpine meadow and alpine steppe.

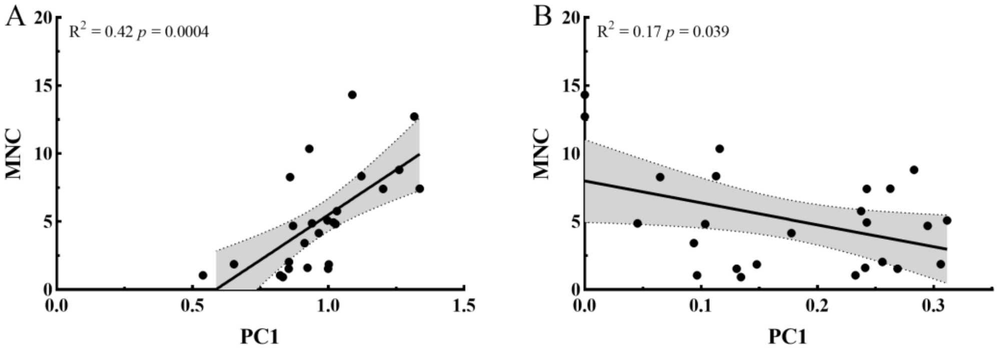

Fig. S3 The correlation analysis of PC1 of dominant (a) and rare (b) microbial communities and microbial necromass carbon (MNC).

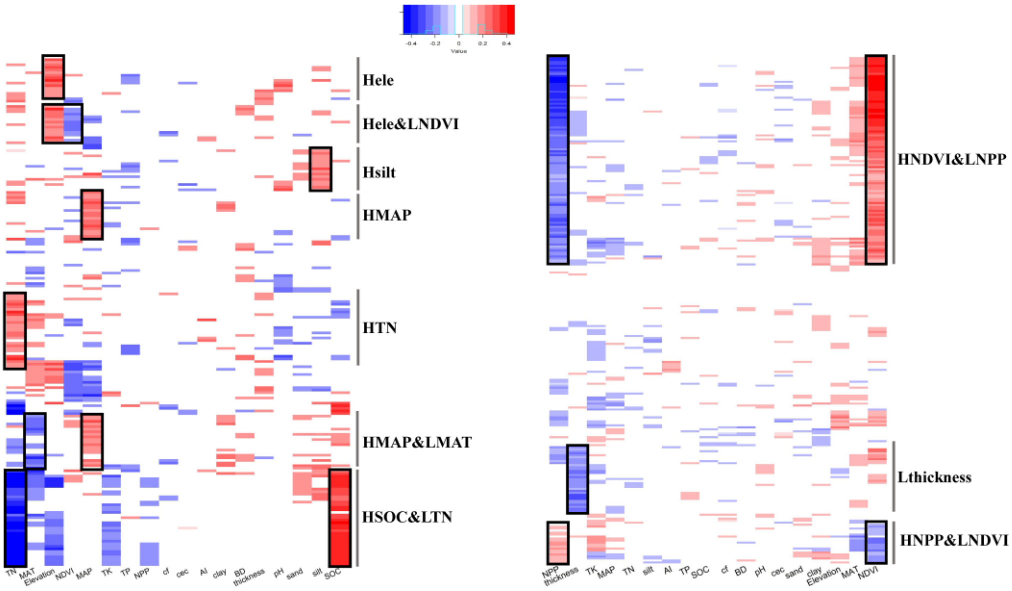

Fig. S4 Heatmap of clustering results for bacteria (a) and fungi (b) eco-clusters. The horizontal coordinates show each environment variable, and the vertical coordinates show each phylotype. The red and blue colors represent positive and negative correlations, respectively. The figure only shows the Spearman correlation between phylotypes and environmental variables with a significance of $p < 0.05$.

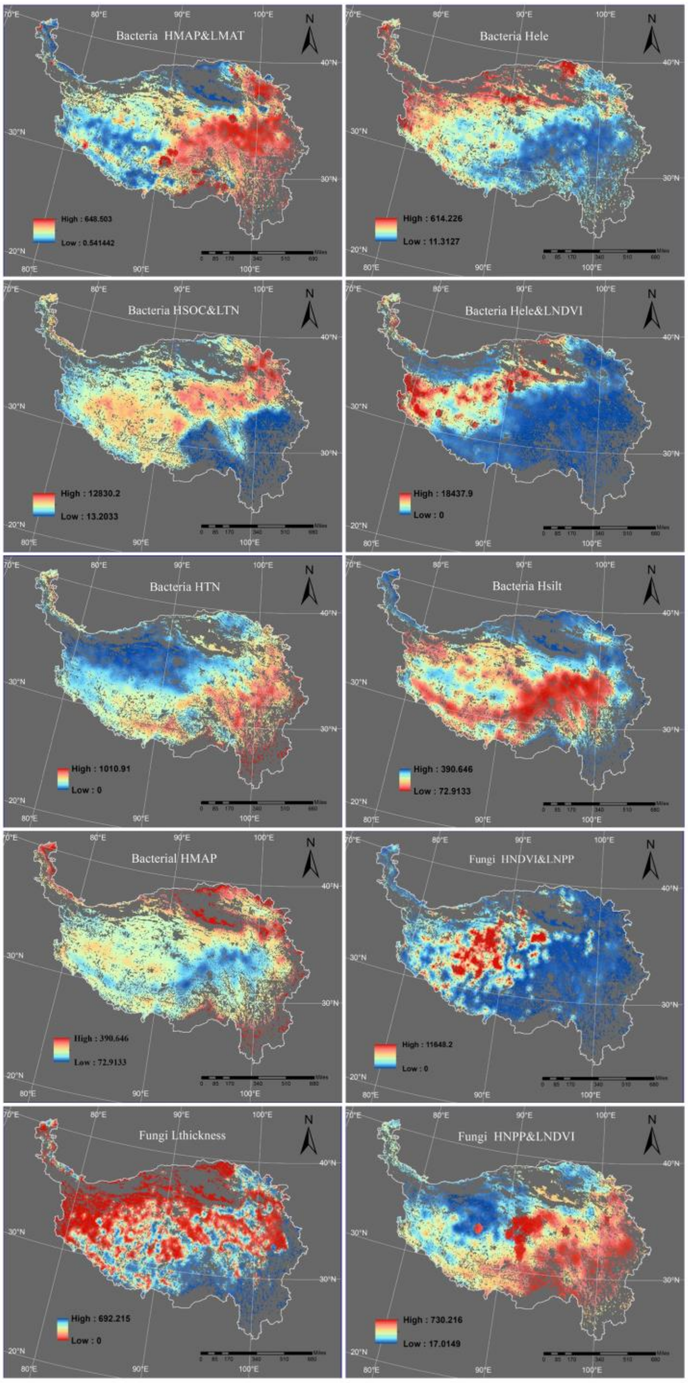

Fig. S5 Projected relative abundance of each bacteria and fungi eco-cluster across the Tibetan Plateau alpine grasslands currently.

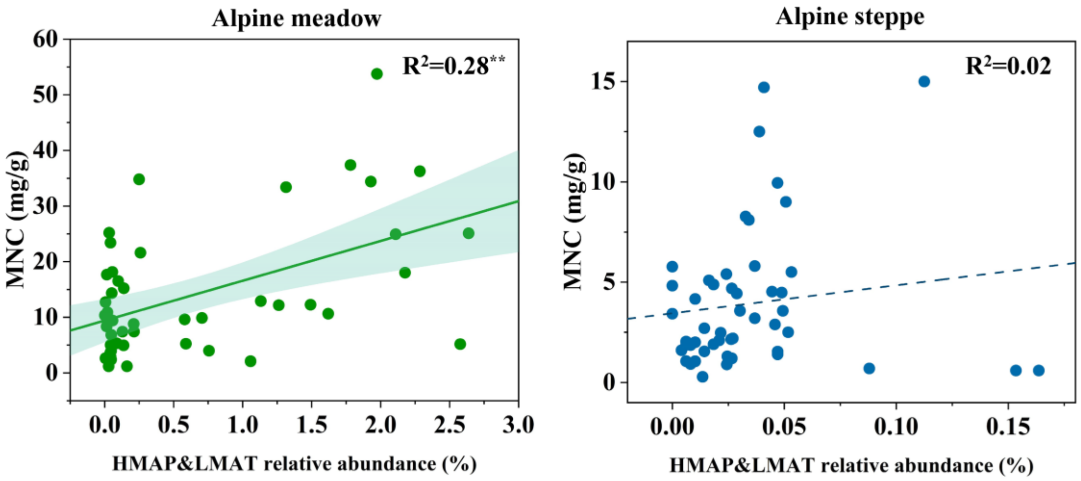

Fig. S6 Relationship between MNC and relative abundance of dominant eco-cluster in two alpine grassland types. The correlation coefficients were shown for significant results. ** indicates $p < 0.01$.

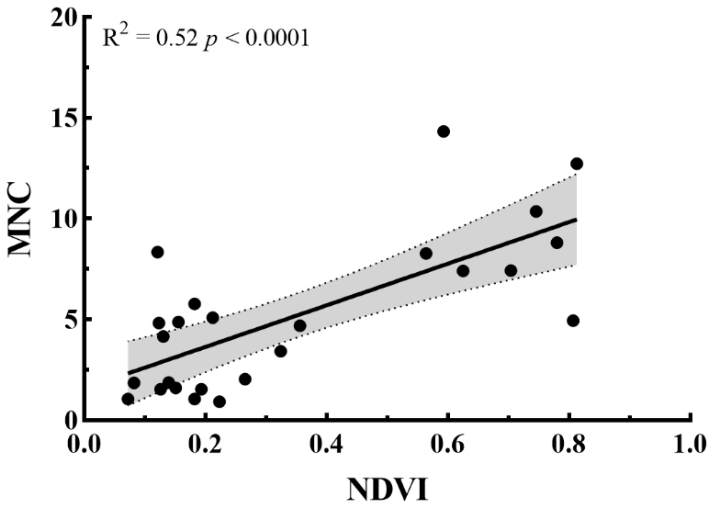

Fig. S7 The correlation analysis of NDVI and MNC.

Table. S1 Simulation performance comparison between the WE and CE model.

	WE model	CE model	D-value
slope	0.74	0.87	0.13 ↑
R^2	0.75	0.82	0.07 ↑
RMSE	4.91	4.16	0.75 ↓
MAE	3.33	2.84	0.49 ↓

Table. S2 Sensitivity of dominant eco-clusters to temperature and precipitation in the Cubist model.

eco-clusters	conds MAT	conds MAP	model MAT	model MAP
HMAP&LMAT	--	--	18%	15%
Hele&LNDVI	2%	56%	32%	65%
Hele	--	--	3%	16%
HSOC&LTN	--	41%	15%	16%
Lthickness	--	--	--	--

conds: conditional sensitivity;

model: projective sensitivity.

Table. S3 Relative abundance of dominant eco-clusters RCP8.5 2050s scenario vs. current.

eco-cluster	future scenario vs. present	test method	p.val
HMAP&LMAT	RCP8.5 2050s vs. current	Mann-Whitney	0.000
Hele&LNDVI	RCP8.5 2050s vs. current	Mann-Whitney	0.000
HSOC&LTN	RCP8.5 2050s vs. current	Mann-Whitney	0.001
