# Supplementary material for: Tibetan Plateau grasslands might increase sequestration of microbial necromass carbon under future warming
Source: Commun Biol. 2024 Jun 4;7:686. doi: 10.1038/s42003-024-06396-y (PMC11150409; doi:10.1038/s42003-024-06396-y)
Supplement: Supplementary file 3 — Reporting Summary [file 42003_2024_6396_MOESM3_ESM.pdf]

nature portfolio

Corresponding author(s): Anzhou Ma

Last updated by author(s): May 28, 2024

Reporting Summary

Nature Portfolio wishes to improve the reproducibility of the work that we publish. This form provides structure for consistency and transparency in reporting. For further information on Nature Portfolio policies, see our Editorial Policies and the Editorial Policy Checklist.

Statistics

For all statistical analyses, confirm that the following items are present in the figure legend, table legend, main text, or Methods section.

n/a	Confirmed	
☐	☒	The exact sample size (*n*) for each experimental group/condition, given as a discrete number and unit of measurement
☐	☒	A statement on whether measurements were taken from distinct samples or whether the same sample was measured repeatedly
☐	☒	The statistical test(s) used AND whether they are one- or two-sided<br>*Only common tests should be described solely by name; describe more complex techniques in the Methods section.*
☐	☒	A description of all covariates tested
☐	☒	A description of any assumptions or corrections, such as tests of normality and adjustment for multiple comparisons
☐	☒	A full description of the statistical parameters including central tendency (e.g. means) or other basic estimates (e.g. regression coefficient) AND variation (e.g. standard deviation) or associated estimates of uncertainty (e.g. confidence intervals)
☐	☒	For null hypothesis testing, the test statistic (e.g. *F*, *t*, *r*) with confidence intervals, effect sizes, degrees of freedom and *P* value noted<br>*Give P values as exact values whenever suitable.*
☒	☐	For Bayesian analysis, information on the choice of priors and Markov chain Monte Carlo settings
☐	☒	For hierarchical and complex designs, identification of the appropriate level for tests and full reporting of outcomes
☐	☒	Estimates of effect sizes (e.g. Cohen's *d*, Pearson's *r*), indicating how they were calculated

Our web collection on statistics for biologists contains articles on many of the points above.

Software and code

Policy information about availability of computer code

Data collection	The observation of MNC from 71 samples were obtained from seven papers by searching for the terms “Tibetan Plateau”, “amino sugar” and “microbial residue carbon” on the Web of Science (http://apps.webofknowledge.com/). The remaining 147 samples were collected along a transect spanning ~3500 km across the Tibetan Plateau during 2013-2014, and the specific sampling method has been described by Ding et al. The climatic variables (MAT and MAP) were obtained from the WorldClim database (http://www.worldclim.org) for 1982-2016, and the aridity index (AI) was obtained from the CGIAR-Consortium for Spatial Information (CSI) GeoPortal (https://cgiarcsi.community) for the period 1970-2000. The plant variables (NPP and NDVI) were downloaded from the National Aeronautics and Space Administration (NASA) Earth Observations website (NEO,https://neo.sci.gsfc.nasa.gov/), and we selected the average NPP and NDVI for the sampling year. The other soil property indicators, including total potassium (TK), silt concentration (silt), clay concentration (clay), sand concentration (sand), gravel concentration (cf), soil bulk density (BD), soil thickness (thickness), and cation exchange capacity (cec) were obtained from the National Earth System Science Data Center, National Science & Technology Infrastructure of China (http://www.geodata.cn).
Data analysis	All of the sequence analyses were performed using the Galaxy pipeline (http://mem.rcees.ac.cn). Ecological clusters and statistical analysis of data used R. Model structure and validation used Python.

For manuscripts utilizing custom algorithms or software that are central to the research but not yet described in published literature, software must be made available to editors and reviewers. We strongly encourage code deposition in a community repository (e.g. GitHub). See the Nature Portfolio guidelines for submitting code & software for further information.

Data

Policy information about availability of data

All manuscripts must include a data availability statement. This statement should provide the following information, where applicable:

- Accession codes, unique identifiers, or web links for publicly available datasets
- A description of any restrictions on data availability
- For clinical datasets or third party data, please ensure that the statement adheres to our policy

All DNA sequencing data in this study was submitted to the Science Data Bank (https://cstr.cn/31253.11.sciencedb.06531; DOI:10.57760/sciencedb.06531.), and is publicly available. All other data are available from the corresponding author upon reasonable request.

Research involving human participants, their data, or biological material

Policy information about studies with human participants or human data. See also policy information about sex, gender (identity/presentation), and sexual orientation and race, ethnicity and racism.

Reporting on sex and gender	No
Reporting on race, ethnicity, or other socially relevant groupings	No
Population characteristics	No
Recruitment	No
Ethics oversight	No

Note that full information on the approval of the study protocol must also be provided in the manuscript.

Field-specific reporting

Please select the one below that is the best fit for your research. If you are not sure, read the appropriate sections before making your selection.

☐ Life sciences ☐ Behavioural & social sciences ☒ Ecological, evolutionary & environmental sciences

For a reference copy of the document with all sections, see nature.com/documents/nr-reporting-summary-flat.pdf

Ecological, evolutionary & environmental sciences study design

All studies must disclose on these points even when the disclosure is negative.

Study description	We conducted sampling across a span of ~20° longitude and estimated the MNC by measuring amino sugars. To determine the climatic, plant-related, and soil physicochemical indexes of the samples, we used a combination of laboratory measurements and an environmental factor database. 16S rRNA gene data was used to predict the relative abundances of the dominant eco-clusters.
Research sample	The site coverage was relatively uniform, including 46 meadow and 50 steppe sample sites.
Sampling strategy	We collected samples between 2019 and 2021 from west to east across about 20° of longitude in the Tibetan Plateau.
Data collection	We collected samples between 2019 and 2021 from west to east across about 20° of longitude in the Tibetan Plateau. The remaining 147 samples were collected along a transect spanning ~3500 km across the Tibetan Plateau during 2013-2014.
Timing and spatial scale	10 years, Tibetan Plateau grasslands.
Data exclusions	No.
Reproducibility	Three.
Randomization	Six.
Blinding	No.

Did the study involve field work? ☒ Yes ☐ No

Field work, collection and transport

Field conditions	No.
Location	About 20° of longitude in the Tibetan Plateau.
Access & import/export	About 20° of longitude in the Tibetan Plateau, 25 sites, 2019-2021.
Disturbance	No.

Reporting for specific materials, systems and methods

We require information from authors about some types of materials, experimental systems and methods used in many studies. Here, indicate whether each material, system or method listed is relevant to your study. If you are not sure if a list item applies to your research, read the appropriate section before selecting a response.

Materials & experimental systems

n/a	Involved in the study
☒	☐ Antibodies
☒	☐ Eukaryotic cell lines
☒	☐ Palaeontology and archaeology
☒	☐ Animals and other organisms
☒	☐ Clinical data
☒	☐ Dual use research of concern
☒	☐ Plants

Methods

n/a	Involved in the study
☒	☐ ChIP-seq
☒	☐ Flow cytometry
☒	☐ MRI-based neuroimaging

Plants

Seed stocks	No.
Novel plant genotypes	No.
Authentication	No.
